# Learning to anticipate mate presence shapes individual sex roles in the hermaphroditic pond snail, *Lymnaea stagnalis*

**DOI:** 10.1007/s10071-022-01623-7

**Published:** 2022-05-06

**Authors:** Beatriz Álvarez, Joris M. Koene, Karen L. Hollis, Ignacio Loy

**Affiliations:** 1 Departamento de Psicología, University of Oviedo, 33003 Oviedo, Spain; 2 Department of Ecological Science, Faculty of Science, Vrije Universiteit, Amsterdam, The Netherlands; 3 Mount Holyoke College, South Hadley, USA

**Keywords:** Biological function, Classical conditioning, Conditioned mating, Mate choice, Mollusc, Pavlovian conditioning, Pulmonate, Sex role conflict, Snail, Simultaneous hermaphrodite

## Abstract

Despite being simultaneously male and female, hermaphrodites may still need to assume the male or female sexual role in a mating encounter, with the option to swap roles afterwards. For the great pond snail, *Lymnaea stagnalis,* deciding which sexual role to perform has important consequences, since sperm transfer and male reproductive success can be decreased. We hypothesised that detecting cues that indicate a possible mating encounter could help them to adapt their mating behaviour. Therefore, we experimentally assessed whether signalling the presence of a conspecific with an odour can affect the sexual role of *Lymnaea stagnalis*. The results showed that learning resulted in either an increased ability to mate as a male or in faster mating compared to the control group. These findings reveal that learning shapes the mating dynamics of *Lymnaea stagnalis*, thus showing that cognitive processes not only affect mating in separate-sexed species but also in hermaphrodites.

## Introduction

Pavlovian conditioning, also referred to as classical conditioning, is a simple type of learning that allows animals to predict the occurrence of relevant events for their survival, such as the availability of food, the presence of predators or even mating opportunities (Domjan 2005). For example, mating encounters can be predicted when a neutral stimulus (i.e. a conditioned stimulus or CS) is reliably followed by the presence of a possible mate (i.e. an unconditioned stimulus or US). This type of learning has been reported to be of special importance for the reproductive success of animals like the blue gourami fish (*Trichogaster trichopterus*) and the Japanese quail (*Coturnix coturnix japonica*). In both species, learning about a CS that signalled the presence of a sexual partner resulted in faster mating and also in greater paternity success as measured by the number of offspring produced (Adkins-Regan and MacKillop 2003; Domjan et al. 1986; Hollis et al. 1997; Mahometa and Domjan 2005).

In cases in which animals can reproduce either as male or female (simultaneous hermaphrodites), mating decisions can be quite complex. For example, in the great pond snail *Lymnaea stagnalis,* a simultaneous hermaphroditic species that mates unilaterally, which means that individuals can perform only one sexual role within a mating interaction, they have to choose which sexual role they are going to perform. In other words, they must gauge their reproductive success based on that particular mating opportunity. Whether *Lymnaea stagnalis* mates in the male role (hereafter also referred to as “sperm donor”) or the female role (hereafter also referred to as “sperm recipient”) has been shown to depend on several variables, including the availability of seminal fluid in the prostate gland (see Nakadera and Koene 2013 for a review). When seminal fluid is available, snails show an increased probability to mate in the male role, and when it is less available, they are more likely to be sperm recipients (Van Duivenboden and Ter Maat 1985).

The above situation can lead to three different mating outcomes within a mating pair: both snails are eager to mate as sperm donors; only one of them is motivated to perform the male role; or, neither is willing to perform that role (in this latter case, mating will not occur). In the first case, that is, when both partners´ prostate glands are full, and hence their propensity to mate as sperm donors is high, they can still gain male and female reproductive success because they can swap sexual roles and mate again after the one mating interaction. However, the snail that mates first as the sperm donor achieves greater male reproductive success than the one that performs the female role first. This difference in the reproductive success between the sperm donor and the sperm recipient is mediated by two known physiological mechanisms with which snails of this species are endowed. The first one is the presence of (at least) two identified seminal fluid proteins (LyAcp5 and LyAcp8b) that affect the performance in the subsequent male function of the sperm recipient (Nakadera et al. 2014): Inseminated individuals can transfer less than half the amount of sperm to their next partner, compared to snails that have not been recently inseminated, which reduces their paternity (Nakadera et al. 2014). It is hypothesised that the snail that mates first as the sperm donor could be gaining two possible benefits: by reducing its partner’s male function, it may be enhancing (1) its own male function because it potentially reduces sperm competition as well as (2) its own female reproductive success, because after mating in one role they can swap sexual roles to mate again and, in such a secondary mating, the first sperm donor might avoid receiving excessive amounts of sperm and unwanted substances such as seminal fluid proteins (Nakadera et al. 2014). The other physiological response that makes it more beneficial to mate first as a sperm donor is that sperm transfer can induce the recipient to allocate more reproductive resources to the female function. As shown in other studies, another seminal protein (LyAcp10) causes a delay in egg laying (Koene et al. 2010), which seems to be related to a higher investment per egg by the recipient (Hoffer et al. 2017). The higher investment per egg would potentially increase the donor’s overall reproductive fitness since its offspring would be expected to be more likely to survive if more resources have been allocated to those eggs (Hoffer et al. 2017). In summary, in a mating encounter between two snails that are motivated to mate as sperm donors, performing the male role first would seem to be the best reproductive strategy, as receiving sperm seems quite costly for both the male and female function of the snail acting as a sperm recipient.

In the situation in which there is a conflict over sex role performance—that is, sperm and accessory gland proteins are available in the prostate gland of both snails—being able to predict a mating opportunity would allow these animals to adapt their mating behaviour so that they can reduce their reproductive costs and/or increase the benefits they may obtain. When only one of the snails within a pair is motivated to mate as a sperm donor, no conflict over sex role performance would be expected. This latter situation could be considered as being functionally equivalent to a mating opportunity in animals that belong to separate-sexed species, like blue gourami or Japanese quail. Previous results have shown that classical conditioning results in faster mating in separate-sexed species, so one might predict that in the absence of conflict over sex role performance, pond snails should also still be able to benefit from conditioning and mate more quickly. However, whether classical conditioning can have an effect on mating, or even whether it has different effects depending on the motivational state of the animals, has never been tested in a hermaphroditic species.

Therefore, the aim of this study was to assess whether classical conditioning can shape the mating dynamics of *Lymnaea stagnalis* and to test whether that effect is unique to the situation in which there is a conflict over the sexual role to be performed*.* Because the mating strategy of this species varies depending on their level of motivation to mate in the male role, the effect of classical conditioning was tested under two different circumstances: one in which there was a conflict over male role performance (hereafter referred to as “Role Conflict”) and another one in which such a conflict was absent (hereafter referred to as “No Role Conflict”). To test this hypothesis, in the first situation, target snails were isolated for a long period of time (14 days), whereas snails used as the US were isolated for a short period (4 days) before mating. Given the benefits of performing the male role in the first place but also the different motivational levels of the two individuals, a mild conflict over performing this role was expected to ensue. Snails in the learning treatment were hypothesised to rely on the predictive CS and, thus, be more likely to mate first as males compared to the snails in the control group.

In the second scenario, in which there was no conflict over sex role performance because one of the snails had already mated as a sperm donor (i.e. the prostate gland was virtually depleted), learning was expected to provide different advantages. As for the blue gourami and Japanese quail mentioned above, conditioning was expected to reduce the total amount of time invested in mating. To test this possibility, the same experiment was conducted again except that the snails employed as USs were kept in groups; that is, they were not isolated prior to the test to keep their motivation to mate as sperm donors at a minimum level.

## Methods

### Subjects

Subjects were sexually mature adult snails (four months old) obtained from the laboratory culture of the Vrije Universiteit in Amsterdam. Snails were housed, either individually or collectively, in plastic pots (measuring 85 × 100 × 80 mm) that were allocated in the same large laminar-flow tank with running low-copper water at 20ºC in the absence of any substrate. Each plastic pot had 22 slits (i.e. 11 on two opposing sides) that allowed for water exchange. They were kept under a 12-h light schedule, starting at 7 am. All snails were given daily access to food (Butterhead lettuce, *Lactuca sativa*) ad libitum and were marked with nail polish on their shell for identification purposes. Snails were randomly divided into two groups: Pavlovian (hereafter referred to as PAV) and control (hereafter referred to as CON) groups. Snails belonging to the PAV and CON groups were isolated 10 days prior to the start of the experiment to increase their motivation to mate in the male role (according to previous studies, De Boer et al. 1996, and 8 days of isolation result in complete replenishment of the prostate gland). Snails were matched by their body size because this variable has also been shown to influence the preference for mating in either the male or the female role (Nakadera et al. 2015). Additionally, other snails were used as the unconditioned stimulus (US). Snails employed as USs were also matched by size.

In the Role Conflict experiment, subjects were 40 sexually mature adult snails (PAV, *N* = 20 and CON, *N* = 20). Another 40 snails were used as USs. Snails belonging to PAV and CON groups had a mean size of 29.46 mm (SEM = 0.24). The snails employed as USs were kept in groups of five snails until the start of the experiment and they had a mean body size of 29.10 mm (SEM = 0.16). From Day 1 onwards, they were kept isolated so that on Day 4 (when the test took place) they had been isolated for 4 days. This isolation period was chosen because the motivation to mate as a male is slightly increased (after 8 days their prostate gland is full) but they still are willing to mate as females (e.g. Nakadera et al. 2014).

In the No Role Conflict experiment, subjects were 30 sexually mature adult snails (PAV, *N* = 15 and CON, *N* = 15). Another 30 snails were used as USs. Snails belonging to PAV and CON groups had a mean size of 28.45 mm (SEM = 0.28). Snails employed as USs were kept in groups of five snails throughout the entire experiment and they had a mean body size of 27.88 mm (SEM = 0.15).

### Apparatus and stimuli

Conditioning and testing were conducted in closed pots, placed on a table top, which were of the same characteristics as the home containers except that they did not have slits. The slits of the home containers served to keep water exchange constant throughout the experiment, whereas the absence of slits in the conditioning and testing pots allowed for an optimal view for the experimenter and level of water in which to run the experiments outside the large laminar-flow tank in which snails were kept throughout the experiment (note that closed pots were also necessary to prevent contamination from chemicals from either the CS or other snails that could be present in the water). Following previously developed procedures (Audesirk et al. 1982), the stimulus used as the conditioned stimulus (CS) was amyl acetate, an oily substance that smells like banana (0.004% v/v). The US was the introduction of another snail for two minutes. Note that two minutes are not enough for the display of the entire behavioural mating sequence in this species. Thus, the US was not a mating encounter but just the presence of a conspecific.

### Procedure and experimental setting

The procedure used for this study was adapted from that employed by Audesirk et al. (1982). Training for the PAV group consisted of 15 training trials conducted over a period of 3 days, 5 trials per day, with an intertrial interval of 90 min. Prior to the start of a conditioning trial, snails were individually transferred from their home tank to a plastic pot, situated on a table top in the same laboratory space, which had been filled with fresh running low-copper water at 20ºC and with no food available. They were left undisturbed for 10 min so they could acclimatise to the new environment.

### Training

**Fig. 1:**
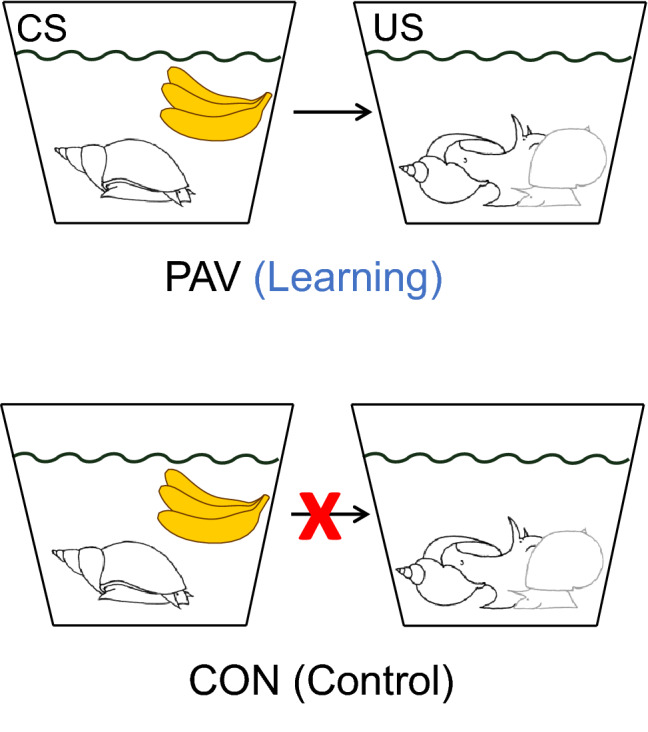
Depiction of the procedure used for conditioning in *Lymnaea stagnalis*. Snails in the PAV (or learning) group were exposed to a solution of amyl acetate (that is, banana essence) followed by the presentation of a conspecific. For the CON (control) group these two events never occurred together as indicated by the red X

Following this acclimation period, snails in the PAV group were exposed to the CS for 2 min. The CS consisted of the presentation of 50 ml of a 0.004% solution of amyl acetate that was gently poured into the plastic jar. The CS was distributed throughout the pot while pouring but not directly on the snails. Subjects were allowed to move around in the solution for 2 min, after which a US snail was introduced into the pot. Both snails were allowed to move around together for 2 min (see Fig. 1 for a visual representation of the protocol). After training, target PAV subjects were individually and briefly transferred for 30 s to a rinsing plastic pot containing clean water, and then to another clean training pot where they waited for the next trial. All the pots in which conditioning and rinsing took place were thoroughly cleaned, so that all the smells were removed and filled again with fresh water. As in Audesirk et al. (1982), this procedure was repeated five times per day, over three consecutive days. Thus, snails in PAV groups received a total of 15 conditioning trials. The intertrial interval was 90 min. The snails used as a US were also placed in a rinsing pot for 30 s before being transferred to a clean new pot. All the US snails were presented to all the target snails (PAV and CON) so the target snails had equivalent experience with the US snails. For snails belonging to the CON group, the treatment was the same except that the CS and the US were never presented together. Rather, they were given unpaired pseudorandom presentations of the CS and the US, i.e. no more than two consecutive presentations of the same stimuli were allowed. As in the PAV group, animals were exposed to 15 presentations of the CS and 15 presentations of the US. To equate for the experience with the two types of stimulation while keeping the level of sexual deprivation equal in both groups, the intertrial interval for the CON group was, therefore, 45 min. The length of CS and of US presentations was also 2 min, as in the PAV group.

### Test

Conditioning was tested by presenting the CS to each target animal for 2 min, after which a US snail was introduced inside the pot. Animals were allowed to interact with each other and mate. The test lasted for 5 h in which the experimenter recorded the behavioural mating sequence every 5 min. The behaviours recorded were mounting, circling, positioning, partial eversion, full eversion, probing, and intromission (see Table 1 for a description of each of them; see also De Boer et al. 1996, p. 168 and 169 for a description and illustrations of the mating sequence).

**Table 1:** Description of the behavioural mating sequence of *Lymnaea stagnalis*

Behaviour	Description
Mounting	Snail climbs on top of the other snail’s shell
Circling	Snail starts to describe circles in a counter clockwise fashion on top of the other snail’s shell to reach the tip of the partner’s shell
Positioning	Snail stops circling on the right edge of the other snail’s shell opening and remains motionless for at least 10 s
Partial Eversion	Snail’s preputium becomes visible but is not fully everted
Full Eversion	The preputium is fully everted
Probing	Snail searches for the female opening with the preputium fully everted
Intromission	Snail introduces the penis (everted from the end of the preputium) into the female gonopore of the partner

These behaviours were recorded for both the target subjects, which were more likely to perform the male role given that they had been isolated for 10 days prior to the start of the experiment (i.e. 14 days before the test) and the US subjects, which were more likely to perform the female role since they had been isolated for only 4 days before the test in the Role Conflict experiment or had not been isolated before the test in the No Role Conflict experiment. The latency to perform each of the behaviours was calculated. Both PAV and CON groups were tested at the same time and the experimenter was unaware of their previous training history. To facilitate the observation of the mating sequence, experiments were run in replicates of ten subjects each (5 PAV and 5 CON).

## Results

In the Role Conflict experiment, two snails belonging to the control group (CON) and one belonging to the Pavlovian conditioned group (PAV) were excluded from the analysis because they did not mate. One subject from the PAV group died during the training period. Thus, each group consisted of 18 subjects.

**Fig. 2:**
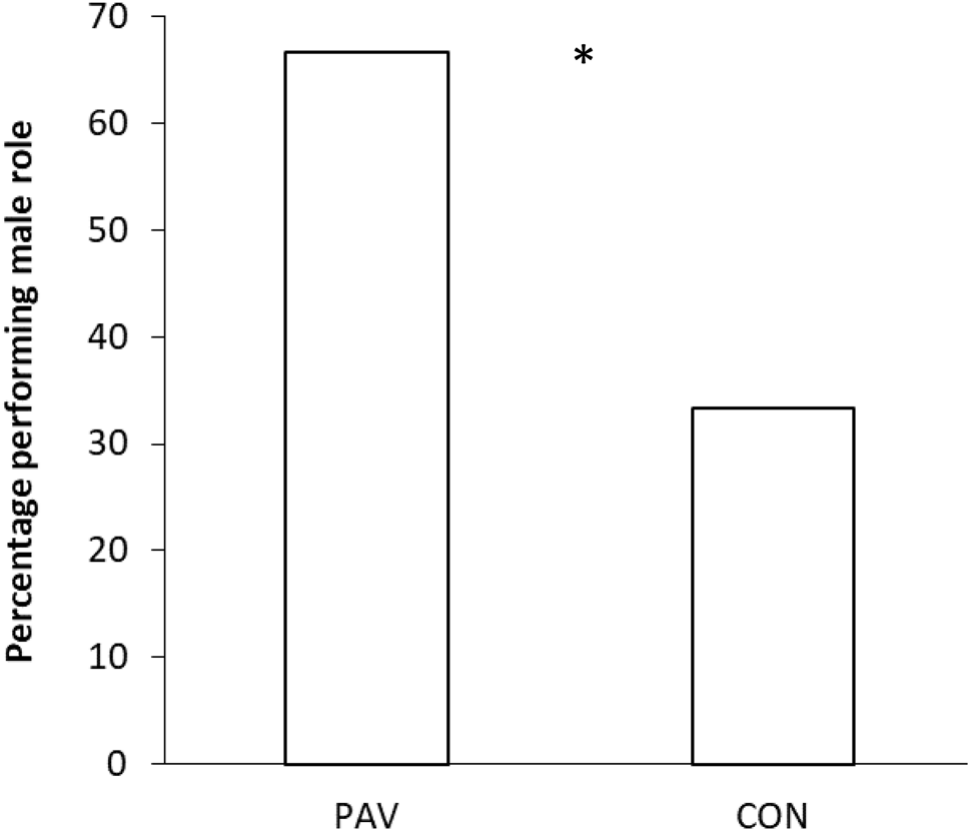
Percentage of PAV (Pavlovian conditioned) and CON (control) subjects that mated first in the male role in the Role Conflict experiment. Statistically significant differences are indicated by asterisks

A larger percentage of the PAV snails that were exposed to a conspecific signalled by an odour mated first in the male role (67%) compared to the CON snails that were exposed to both the odour and the conspecific but in an unpaired fashion (33%) (Fig. 2). This difference in their preferred role performance was statistically significant [χ^2^ = 4; *p* = 0.045] and shows that learning to predict the presence of a conspecific increases the likelihood of mating first as a sperm donor in a signalled mating encounter. When looking at the latency to perform each of the steps of the mating sequence, snails in the CON group that mated first in the male role were always slightly, but non-significantly faster than snails in the PAV group [*F*(1,15) < 1.564, *ps* > 0.23, η^2^ < 0.94].

In the No Role Conflict experiment, two snails died during the experiment (one belonging to the PAV group and the other one belonging to the CON group). Out of the 14 subjects remaining, 4 snails in the PAV group and 5 in the CON group did not mate and they were excluded from the analysis. Mating did not occur either because none of the target snails showed any interest in mating or because the snail employed as the US (and thus expected to perform the female role) avoided mating by crawling out of the water.

**Fig. 3:**
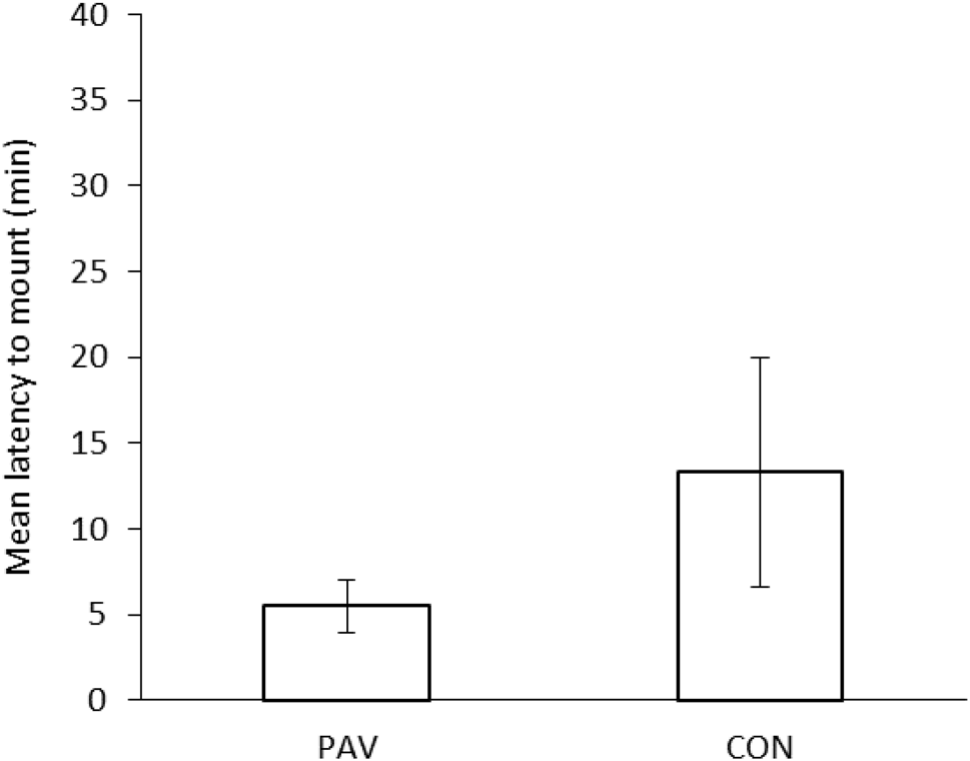
Mean latency of PAV (Pavlovian conditioned) and CON (control) subjects to mount the other snail’s shell in the No Role Conflict. Vertical bars represent SEMs

**Fig. 4:**
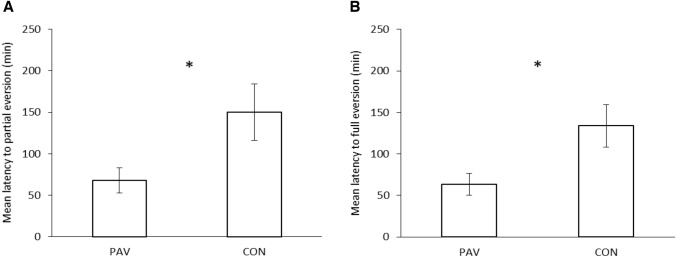
**A** Mean latency of PAV (Pavlovian conditioned) and CON (control) subjects to show partial eversion and **B** to show full eversion in the No Role Conflict experiment. Vertical bars represent SEMs. Statistically significant differences are indicated by asterisks

**Fig. 5:**
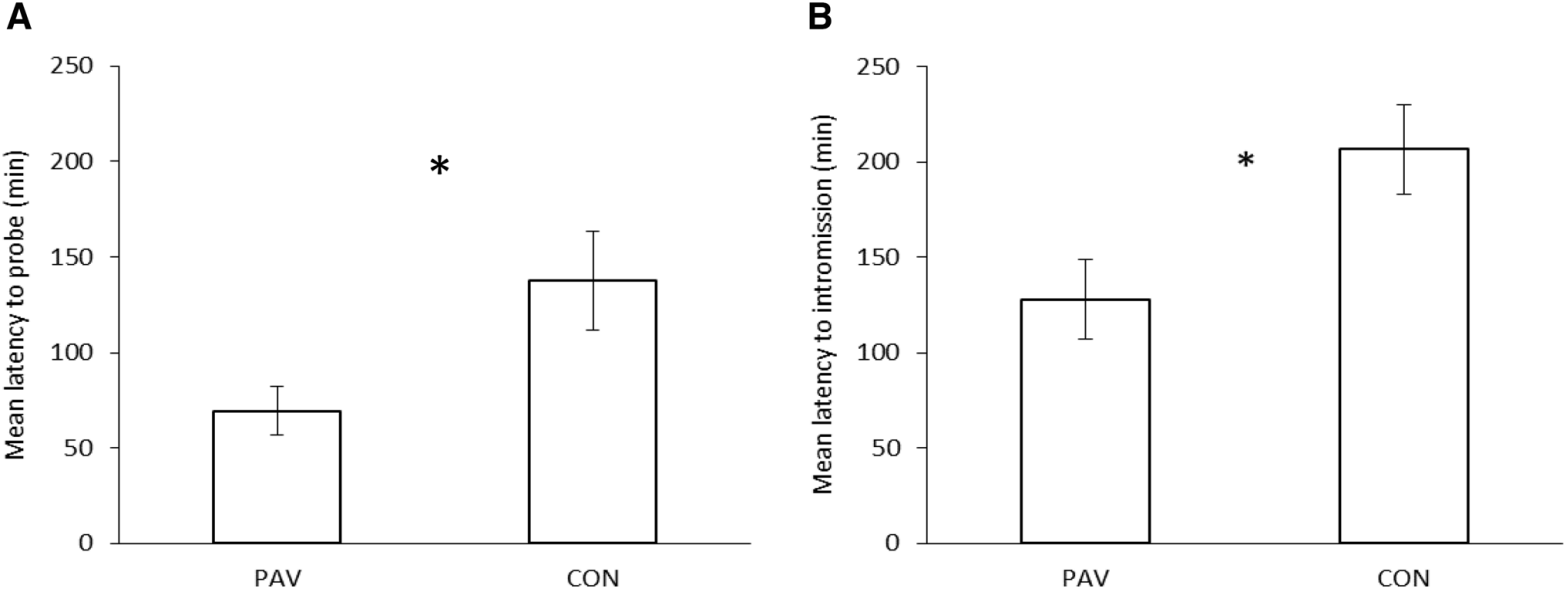
**A** Mean latency of PAV (Pavlovian conditioned) and CON (control) subjects to start probing and **B** to intromit in the No Role Conflict experiment. Vertical bars represent SEMs. Statistically significant differences are indicated by asterisks

As expected based on the difference in isolation times between the focal snails (14 days) and the snails used as USs (no isolated), there was no significant difference in the likelihood of focal snails mating in the male role (i.e. all PAV and CON snails mated as males). Snails in the PAV group, which were exposed to an odour cue (a solution of amyl acetate) followed by the introduction of another snail, showed a shorter latency to mate compared to the CON snails that had been exposed to both stimuli, but in a pseudorandom fashion. Although the difference in latency was not significant for the behavioural components at the beginning of the mating sequence, namely mounting (Fig. 3), circling and positioning [*Fs*(1,17) < 1.766, *ps* > 0.201, η^2^ < 0.094], statistically significant differences in latency were found for the behaviours displayed at the end of the mating sequence: partial [*F*(1,17) = 5.318, *p* = 0.034, η^2^ = 0.238] and full eversion [*F*(1,17) = 6.364, *p* = 0.022, η^2^ = 0.272] (Fig. 4A and 4B, respectively), probing [*F*(1,17) = 5.874, *p* = 0.027, η^2^ = 0.257] and intromission [*F*(1,17) = 6.271, *p* = 0.023, η^2^ = 0.269] (Fig. 5A and 5B, respectively).

## Discussion

The aim of this study was to test the role that classical conditioning could play in a simultaneous hermaphroditic species that needs to decide not only on whether to mate or not, but also on which role to perform. Animals in PAV groups were exposed to paired presentations of an odour cue followed by the presence of a conspecific, and animals in the CON group were exposed to the same stimulation except that these two events never occurred together.

In the Role Conflict experiment, a medium level of conflict over male role performance was arranged by differentially isolating the target and the US snails prior to the test. The results obtained showed that classical conditioning led to an increased male role mating performance for the PAV group compared to the CON group. Importantly, focal snails of the PAV and the CON group had been isolated for 14 days (which means that their prostate gland was virtually full as it takes them about 8 days to replenish it), whereas snails used as the US had been isolated for only 4 days (i.e. their prostate gland was virtually half full). Thus, although under such circumstances, focal snails of both groups were expected to be more likely to mate first in the male role (van Duivenboden and Ter Maat 1985), the fact that significantly more did so in the PAV group than the CON group shows that conditioning had an important impact on this mating decision, and that sex role performance is not solely determined by physiological mechanisms (i.e. availability of seminal fluid). In other words, the mating response is not simply a function of the accumulated sperm, but it is also shaped by previous experience. Here, we would like to emphasise that such a mating decision does not need to be conscious, and that we use the word “decision” in agreement with previous literature on sex role performance in *Lymnaea stagnalis* (see e.g. Nakadera et al. 2015), and in the sense that there are several available options (mate in the male role, mate in the female role or avoid mating) among which the snails are deciding.

In the No Role Conflict experiment, no differences were observed in the frequency of snails performing the male role. This was expected given that the snails used as USs had not been isolated prior to the mating encounter, so their prostate gland was most likely depleted and they were, thus, not inclined to mate as sperm donors. However, classical conditioning did have an effect on the time invested in performing the complete mating sequence, shortening the overall time to reach intromission.

Taken together, the results of this study show that *Lymnaea stagnalis* can learn to predict a mating encounter and that mating behaviour is affected by simple forms of learning such as classical conditioning. The reduction of total amount of time invested in mating that was observed in the No Role Conflict experiment is in accordance with the results of previous studies with other species, such as blue gourami (e.g. Hollis et al. 1997) and Japanese quail (e.g. Domjan et al. 1986), in which it was also shown that signalling the presence of a possible partner resulted in faster mating. However, as shown in the Role Conflict experiment, classical conditioning can also affect the mating strategy at a higher level, as indicated by the preference shown by the animals in the learning condition to mate in the male role. As described above, mating in the male role first potentially provides greater benefits for these hermaphrodites, because they can avoid costs induced by received seminal fluid proteins, so in the particular situation in which there is a conflict over sex role performance, being able to predict a mating opportunity seems to be of great advantage for this species. Learning would be expected to boost the reproductive success of the snails when mating as sperm donors first, as it would avoid reduction of the male reproductive function via the seminal fluid proteins that have been reported to reduce the partner’s sperm transfer and to change the partner’s egg investment (e.g. Nakadera and Koene 2013). Furthermore, because the only difference between the two experiments was the level of motivation of the snails used as the US to mate as sperm donors, this study indicates that snails of this species are somehow able to detect the motivational status of their partner to adapt their mating strategy accordingly. It also illustrates that classical conditioning does not simply imply the display of a fixed behavioural pattern. Rather, it allows animals to confront different situations in a flexible way. Classical conditioning thus allows these freshwater snails to predict a mating encounter and to adapt their behaviour accordingly, so that they can potentially obtain more benefits.

As argued in previous studies (e.g. Hollis et al. 1997), it could be possible that animals in the control group showed a lower rate of male performance in the Role Conflict experiment or took longer to mate in the No Role Conflict one because they had actually learned that the CS signalled the absence of the US. In other words, since the CS and the US were explicitly unpaired, they could have acquired inhibitory learning that would have resulted in a slower response capacity in the test. Although the occurrence of inhibitory learning in this particular study has not been tested, previous research has shown that the explicitly unpaired group serves as a good baseline against which both classical conditioning and its biological function can be tested (see e.g. Audesirk et al. 1982 and Hollis et al. 1997, respectively). Thus, the results here reported are most likely solely due to the excitatory learning acquired by the animals in the learning condition. Nonetheless, if inhibitory learning was to occur in the control group, such results would not question the main finding of this study, that is, that learning shapes the mating strategies in this hermaphroditic species.

In this study, we have focused on the effect of learning on the snails´ performance as sperm donors, but it remains to be analysed whether it could also affect the sperm recipient’s response or its reproductive success. For instance, *Lymnaea stagnalis* snails can actively avoid mating in the female role, especially when they have been recently inseminated (Moussaoui et al. 2018; Daupagne and Koene 2020), and, as already mentioned, receiving sperm and seminal proteins seems quite costly for both the male and the female functions of the sperm recipient. In this regard, it would be interesting to test whether Pavlovian conditioning results in the sperm recipient to be more likely to avoid mating or if, as in separate-sexed animals, it can facilitate the female mating response (e.g. Gutiérrez and Domjan 1997, 2011). Furthermore, quantifying the effects of learning in terms of paternity success would also help to better understand to what extent cognition can shape the physiological responses of hermaphroditic animals in a mating situation and if the effect can be comparable to that already observed in blue gourami (e.g. Hollis et al. 1997) and Japanese quail (e.g. Adkins-Regan and MacKillop 2003). In this regard, *Lymnaea stagnalis* constitutes a perfect model for such analyses, not only for it being a simultaneous hermaphrodite that still allows for exploring the male and the female reproductive functions separately, but also because much of its reproductive physiology, mating behaviour, cognition and neuroscience has already been investigated in detail (Fodor et al. 2020). Although much research remains to be conducted, this study lays the foundation for future ones as it constitutes the first demonstration of the adaptive effects of conditioned mating in a hermaphroditic species.

To conclude, most importantly, the results demand the recognition of learning as a cognitive process that affects mating not only in separate-sexed species, but also in hermaphrodites. Mating has been regarded as a simple behavioural response or action that animals display automatically or innately (e.g.Andersson and Simmons 2006; Kopp et al. 2018). However, there is growing evidence that cognitive processes such as learning or decision making are greatly involved in each mating encounter (e.g. see reviews Alvarez and Koene 2018; Bateson and Healy 2005; Pfaus et al. 2001; Ryan 2021). The results obtained in this study have served to broaden our understanding of learning and its modulatory effect on mating as they show that cognitive processes also affect mating decisions in hermaphrodites.
